# Psychometric Properties under EFA, CFA, Measurement Invariance, and IRT Models for Older Adults' First Aids Knowledge Scale among Iranian Grandparents: The Modified Scale

**DOI:** 10.1155/2024/6208571

**Published:** 2024-08-26

**Authors:** Atefeh Rahim, Abdolrahim Asadollahi, Mehdi Mojadam, Eva Dolenc Šparovec, Mansour Kashfi, Mahin Nazari

**Affiliations:** ^1^ Students Research Committee Shiraz University of Medical Sciences, Razi Ave., Shiraz, Iran; ^2^ Department of Gerontology School of Health Shiraz University of Medical Sciences, Razi Ave., Shiraz, Iran; ^3^ The Middle East Longevity Institute, Azmi Street, Abdo Center, P.O. Box: 618, Tripoli, Lebanon; ^4^ Health in Disasters and Emergencies Department of Public Health School of Health Abadan University of Medical Sciences, Abadan, Iran; ^5^ Public Health Division Faculty of Health Sciences University of Ljubljana, Zdravstvena Pot 5, Ljubljana 1000, Slovenia; ^6^ Department of Public Health School of Health Shiraz University of Medical Sciences, Razi Ave., Shiraz, Iran; ^7^ Department of Health Promotion School of Health Shiraz University of Medical Sciences, Razi Ave., Shiraz, Iran

## Abstract

This research aims to create and evaluate an assessment tool termed Older Adults' First Aid Knowledge Scale, which measures the knowledge and attitude of Iranian grandparents about first aid. In accordance with COSIM guidelines, 485 individuals in southern Iran completed the instrument as part of a psychometric investigation. Rasch partial credit model (PCM), exploratory factor analysis (EFA), confirmatory factor analysis (CFA), and receiver operating characteristic (ROC) analysis were used to analyze the results. The final version of OFAKS consisted of 18 items that were validated through EFA, CFA, and item response theory (IRT) analysis. All items showed measurement invariance and consecutive response groupings in the predictable order, and the instrument had strong internal consistency. Although Rasch's analysis demonstrated the significance of OFAKS, further investigations and testing in different settings are required to confirm the validity of the tool.

## 1. Introduction

With the increasing rate of global aging, the population growth of older adults in some developing countries such as Iran and other Middle Eastern and North African (MENA) countries is 3.3 to 4.8 times greater than the developed ones. The rapid aging of the global population presents a unique challenge in developing countries like Iran, where the older population is growing much faster than in developed nations. Importantly, almost two-thirds of adults live in these developing countries [1]. Accidents and injuries are the leading cause of death in young children, making it a significant concern in social and health policies [2]. Incidents at home account for 35% of unintentional childhood injuries, while for 6-year-old children, this percentage (60–80%) is a significant concern [3]. Accidents that take place within the household are more widespread compared to other forms of accidents. This can be attributed to the continuous presence of potential dangers and the fact that the risk of an accident persists throughout the entire day. Moreover, despite feeling safe and protected at home, it is unfortunate that a significant number of children suffer injuries or even lose their lives within the confines of their own residences [4]. Amongst all age groups, children between the ages of 0 and 6 face the greatest vulnerability to such risks [5].

Child safety remains a top concern among parents today. Growing up, children are vulnerable to injuries and accidents due to their limited ability to respond quickly and appropriately in emergency situations. They also have little control over their surroundings, which further increases the risk of harm and even death. It is crucial for those around the child, especially family members, to be vigilant and take necessary measures to protect children from domestic accidents. Families should be aware of potential hazards in and around the house [5]. To this end, grandparents play a significant economic and social role in providing childcare to families, with 58% of grandmothers and 49% of grandfathers in ten European countries taking care of at least one grandchild under the age of 16 in the past year in the absence of parents [6].

In recent years, there has been a significant increase in the availability of full-time employment options for women, particularly mothers, in Iran. As a result, the importance of grandparents stepping in to care for their grandchildren in the absence of their mothers has gained prominence. This practice has now become deeply ingrained in the child-rearing culture of the Middle East, mirroring similar customs observed in Far Asian countries such as China, Taiwan, South Korea, and Japan. The prevalence of injuries and accidents for children in Iran may pose a challenge, especially in light of the decreasing size of families (nuclear family). Statistical data reveal that among cases under 5 years of age examined by Iran's forensic medicine, 30.4% of deaths are attributed to domestic accidents, including incidents like drowning, burns, and electrocution, while 21.1% are linked to urban road accidents. The prevalence of deaths resulting from accidents and mishaps in both urban and suburban areas underscores the significance of addressing safety measures to prevent such fatalities. A separate study indicates that nontraffic accidents and incidents constitute 12.5% of the causes of death in this age group, with traffic accidents and incidents contributing to 9.8% of fatalities among children under 5 years of age in Iran.

Preventing home accidents can be achieved through simple interventions such as improving the home environment, providing regular training to parents who spend most of their time with children, and increasing parental awareness [5]. Most domestic accidents can be prevented by increasing awareness, improving the home environment and greater safety [7]. Notably, nonprofessionals, such as grandparents, can play a vital role in saving children's lives during emergency situations.

Learning life-saving first aid (LSFA) skills, including first aid (FA) and cardiopulmonary resuscitation (CPR), should be a part of basic health education for all people starting from the age of 10, according to Bollig et al. [8]. Nevertheless, enabling individuals to provide life-saving first aid in a medical emergency requires training [9]. FA is the set of emergency care or treatment given before obtaining regular medical help, and it should be readily available to the general public without hindering professional examination or wound treatment [10]. Due to the increasing number of working parents in today's world, grandparents are now more responsible for the care of their grandchildren. Consequently, they should also be introduced to FA measures, including resuscitation. To achieve this, the first step is to assess the level of knowledge of parents and grandparents in the field of first aid using the FA questionnaire for older people. To this end, the Older Adults' First Aid Knowledge Scale has been translated, developed, and validated in the Persian language.

The OFAKS evaluates middle-aged and older adults' knowledge, attitudes, barriers, and intentions regarding folic acid and its importance. The OFAKS questionnaire (12-items), originally developed in Slovenia, assesses knowledge about first aid among older adults by Dolenc et al. [11]. However, this instrument was only validated in the Slovenian language among 3355 adult inhabitants of Slovenia, and its psychometric properties (i.e., EFA, CFA, and IRT) were not reported in their paper. To address this gap, the current study aimed to develop a Persian version of the OFAKS questionnaire and determine its psychometric properties in Iran.

The increasing number of older people taking care of their grandchildren, combined with more job opportunities for mothers, highlights the importance of being awareness about first aid to prevent accidents and injuries at home. It is crucial for families and the healthcare system to have an effective scale to assess the level of first aid awareness among grandparents.

## 2. Materials and Methods

To validate and modify the OFAKS questionnaire, a cross-sectional psychometric study was conducted at the Farzangan Daily Care Foundation (FDCF), a residence for over 17,500 individuals aged 60 and above located in southern Iran. Based on these parameters, the sample size was calculated to be 485 people. To select the participants for the study, the names of eligible seniors were entered into Excel software, and eligible participants were classified using the inclusion criteria. Each person was assigned a membership ID, and the sample was randomly selected using the random numbers table.

The study's inclusion criteria were individuals aged 60 years or older who desired and provided informed consent to participate, had literacy skills, had grandchildren under five years of age, and took care of their grandchildren in the absence of their parents. Exclusion criteria included failing to answer the questionnaire, suffering from an illness that affected their ability to provide care for the child, unwillingness to continue cooperation, failing to answer more than three questions of OFAKS, and failure to take care of the grandchild. Demographic information such as age, gender, number of hours spent caring for grandchildren in 24 hours, and number of grandchildren was collected for each participant.

### 2.1. Instrumentation

The published version of the OFAKS in English in 2021 includes 12 items, six of which are related to FA knowledge and six of which are related to attitude towards FA. This tool helps measure and identify the knowledge and attitude of older adults towards FA. The main language of the OFAKS was Slovenian in the study by Dolenc et al. [11], whose English version is included in the appendix of the current paper.

The original version of OFAKS was created by a team at the University of Ljubljana in Slovenia in 2021 and was administered over the phone to 3355 Slovenian citizens, 35% of whom were over 60 years old (1174 individuals) [11]. The questionnaire was analyzed with pre- and post-tests and an expert panel, and all items, except for the emergency medical phone number question, had three answer options. The instrument's scoring range was from 0 to 10, with a low score indicating limited knowledge of first aid. While the English version of this tool is available, no validation was found in other languages, and classical EFA and CFA validation and modern IRT models are not reported for this instrument.

### 2.2. Procedure

The research instrument was translated and validated according to the protocol of the World Health Organization (WHO) after obtaining permission from the designers of the OFAKS. Initially, the questionnaire was translated from English to Persian by two independent translators, and a common version of the initial questionnaire was obtained in a meeting attended by the translators. Ten literate older adults possessing at least a bachelor's degree were interviewed to evaluate the face validity of the questionnaire. They were asked to check the level of difficulty, appropriateness, and ambiguity of the items and offer their suggestions to determine the comprehensibility of the questionnaire. After confirming the face validity, the content validity was evaluated using the content validity ratio (CVR) and then the content validity index (CVI). According to the Lawshe table, if ten evaluators are used, the acceptable limit of CVR is 0.62.

During the study, it was found that the validity ratio for certain items, such as 3 and 7, was less than 0.6. In the second session for developing the OFAKS, the test was reviewed with an expert panel. The panel consisted of eight clinical experts and PhD holders in Emergency Medicine, Health In Emergencies and Disaster, Pediatrics, Geriatrics, and Gerontology. They had at least eight years of work experience. The expert panel provided their opinions, and through a semistructured interview and brainstorming, new themes were extracted. Finally, with face and content validity, the OFAKS was developed to include 20 questions with three answer options (0, 1, and 2) and was classified into four subscales: Knowledge (items 1 to 8), Attitude (items 9 to 13), Obstacles/Barriers (items 14 to 19), and Intention (items 19 and 20). The scoring range for each of the subscales includes 0 to 9, 0 to 10, 0 to 10, and 0 to 4, respectively, and the total score range of OFAKS includes 0 to 33. A higher score indicates a better and more accurate measure of FA. The overall CVI was determined to be higher than 0.71. After confirming the validity of the form and content, this version of the questionnaire was presented to 5 literate older adults for reading comprehension. Their feedback was subsequently collected and used to improve the questionnaire.

In the next step, 285 older men and women completed the 20-item version of the OFAKS questionnaire, which was then entered into SPSS software (v. 28). To determine OFAKS' construct validity, EFA with the Maximum Likelihood model, Varimax rotation method, and Scree-Plot were used to identify its factors. Presuppositions related to the adequacy of the samples were checked using the Kaiser-Meir-Olkin (KMO) test and Bartlett sphericity test, which confirmed these assumptions [12]. During the following step, the results of the EFA were calculated on the second group population (*n* = 200) using the confirmatory factor analysis method (CFA) in JAMOVI 2.4.8 software (2023), and the fit indices were measured [13]. Finally, the internal homogeneity of the OFAKS was tested using McDonald's omega coefficient, Cronbach's alpha, and Pearson's correlation [14, 15].

The study utilized the JAMOVI 2.4.8 software to conduct an IRT analysis, specifically using the partial credit model (PCM) and rating scale model (RSM) [16, 17]. The analysis also included measuring the measurement invariance (MI) for good fit estimates across the groups, as well as checking the internal stability of the tool by examining the intracluster correlation index (ICC). Unfortunately, we were unable to determine the cutoff points of OFAKS using receiver operating characteristics (ROC) curve analysis due to the lack of a criterion questionnaire in the Persian language. The study adhered to the Helsinki Convention, the COSMIN checklist, and the STROBE guide and was approved by the Ethics Committee of Shiraz University of Medical Sciences (IR.SUMS.SCHEANUT.REC.1402.009 on March 5, 2023). All participants provided verbal and written informed consent, and data were collected between March and June 2023.

## 3. Results

### 3.1. Participants' Characteristics

The research involved almost 485 older adults, with 56.7% males and 43.3% females. Among them, 93% live with their spouses and 84.7% of them have taken care of at least one grandchild in the past year. Their average age was 63.4 (with an SD of 8.57). The mean score of the OFAKS 20-item scale was 12.91 for all participants, with a range of 0–33, an SD of 4.65, a median score of 12, and an interquartile range of 6.75. For men, the mean score was 13.33 (SD = 4.77, IQR = 7), and for women, it was 12.37 (SD = 4.47, IQR = 6). The reliability coefficient (McDonald's Omega) was 0.645 (Cronbach's *α* = 0.655). Moreover, there were no significant differences between male and female participants in the total score of OFAKS (*t* = 1.255, Cohen's *d* = −0.11 [95% CI = −0.1182–0.5307], df = 483, *P* > 0.05).

### 3.2. OFAKS Validity

The results of the EFA (*n* = 285) and CFA (*n* = 200) performed on the OFAKS are presented in Table 1. Construct validity was tested using Maximum Likelihood (ML) with 11 times varimax rotation. The Kaiser–Meyer–Olkin (KMO) test for sampling adequacy was 0.846 (95% CI = 0.640–0.912), indicating good sampling adequacy, and Bartlett's test for homoscedasticity of variances across the samples was significant (924.4, *P* < 0.05). The study extracted four factors with eigenvalues of 74.11, factor determinacy index (FDI) = 0.937, variance explained = 73.51, and mean score of uniqueness (Psi) measure for 18 items >0.7 (without items 3 and 7). These factors represent the overall propriety of the OFAKS and its four subdomains, namely, knowledge, attitude, barriers, and intention domains. The study found that the internal consistency of most subscales (factors 1 to 4) of the OFAKS revealed good internal consistency as Cronbach's alpha and McDonald's omega (≥0.68), factor determinacy index (≥0.9), and ORION index (≥0.9) [18, 19].

Based on the table above, it can be observed that Factor 1 (Knowledge) includes items 1, 2, 4, 5, 6, and 8, while Factor 2 (Attitude) contains items 9 to 13. Factor 3 (Barriers) comprises items 14 to 18, and Factor 4 (Intention) consists of items 19 and 20. The most commonly used fit indices (RMSEA = 0.44, SRMR = 0.072, CFI = 0.951, GFI = 0.958, and TLI = 0.942) demonstrate that there is an acceptable fit between the hypothetical model and the data. All comparative fit indices showed good model fitness of OFAKS 18-item, *P* < 0.05 [20]. However, items 3 and 7 were removed from the OFAKS' items as they did not meet the criteria of the CFA due to less measure of uniqueness (Psi) (<0.2), as evident in table (*P* > 0.05).

### 3.3. IRT Psychometric Analysis for OFAKS

The Rasch Rating Scale Model (RSM), also known as the polytomous Rasch model, developed by Andrich in 1978, and the Partial Credit Model (PCM) are both models of IRT analysis used for instruments with polytomous items. These models were extracted for the scale (see Table 2). The Infit (information-weighted mean square statistic), Outfit (outlier-sensitive mean square statistic), Delta-tau, and Thurstone thresholds of the PCM all indicate the well and unique distribution of items' threshold in difficulty, person's ability, and discriminant, which are the same across items in the OFAKS (*P* < 0.001) [21]. This indicates that the scales are well designed for respondents (Person Reliability = 0.676, MADaQ3 > 0.882, *n* = 485, *P* < 0.001). Additionally, some fit indices in IRT analysis were satisfactory, such as the item separation index (8.32, suggested cutoff >2), person separation reliability (0.84, suggested cutoff >0.7), item separation reliability (0.91, suggested cutoff >0.7), and person separation index (2.32, suggested cutoff >2) [16, 17]. However, items 3 and 7 of OFAKS, due to high measures in difficulty and a person's ability, seem to be deleted (delta-tau >0.9). In the OFAKS, 18 items confirmed satisfactory fit statistics (ranging from 0.45 to 1.8), not including items 3 and 7, which showed undesirable ones (equal to 3.211 and 2.113).

**Figure 1:**
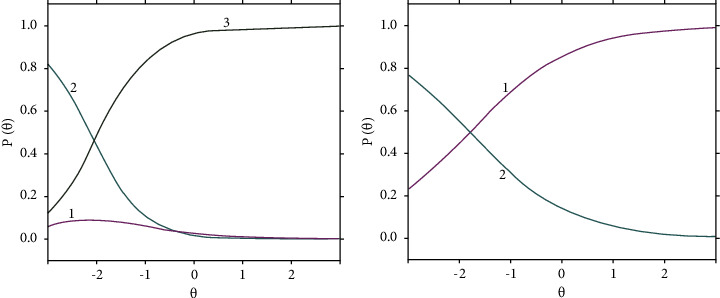
Items 3 and 7 of OFAKS with the lowest fitting among all items.

**Figure 2:**
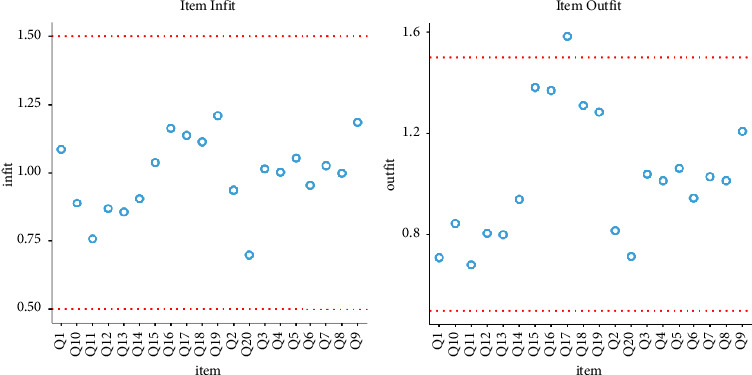
Item infit and outfit plot for OFAKS.

The results of PCM and RSM models are shown in Figures 1 and 2, which illustrate the infit and outfit plots. These figures also illustrate the intersections of the category probability curves (CPC), also known as theta for measuring ability parameters in the IRT context, for two misfitted items, i.e., 3 and 7, as samples, with the remaining items [16, 17].

### 3.4. Measurement Invariance Results

Table 3 displays the results of the MI analyses for older men and women. The results show that the goodness-of-fit was excellent for all MI models, there were minimal differences between the descriptive fit parameters, and the likelihood ratio tests indicate that there were no significant differences in model fit between MI models (with and without menopausal spouse). Therefore, an equal fit across all MI models was obtained for the OFAKS among both samples (ΔSRMR and ΔRMSEA ≤0.05) [22, 23].

### 3.5. OFAKSs (Short-Version) Reliability

The internal consistency of the tool was assessed using Cronbach's alpha, McDonald's omega coefficients, and the intraclass correlation coefficient (ICC). The results of the ICC calculation indicated that the internal consistency of the tool was good, with a value of ICC = 0.71 (*n* = 485, *P* < 0.002, 95% CI = 0.69–0.78). Cronbach's alpha and McDonald's omega were also found to be above 0.65, which is considered an acceptable level of internal consistency. Additionally, Fleiss's kappa coefficient (1981) was calculated as 0.74 in this study, indicating the high reliability of the tool (*P* < 0.002, 95% = 0.68–0.79). Furthermore, Table 4 shows the results of the Pearson correlation coefficient (PCC) between the OFAKS sum score and its domains. The *r*-value of four domains and the total score of OFAKS are significantly high (rp ≥ 0.4, *P* < 0.01 and 0.05, *n* = 485). However, it was not possible to establish the cutoff points of the OAKS using ROC analysis due to the lack of alternative and equivalent questionnaires (criterion-related validity) in the Persian language and golden standard.

## 4. Discussion and Conclusion

The main findings of the current research are as follows: (1) classic factor analysis (EFA & CFA) confirmed that OFAKS consists of 18 items with a 4-factor structure. (2) OFAKS is found to be more reliable when used with Iranian samples. (3) The IRT analysis models (PCM & RSM) also confirmed the 18-item structure of OFAKS. (4) The OFAKS is a practical tool for assessing the first aid knowledge of older adults. (5) The research also evaluated the attitudes and barriers towards participating in first aid education. (6) The OFAKS is used in community-based settings to inform health policy making.

This study assessed the reliability of the 18-item OFAKS scale by using Cronbach's alpha, McDonald's omega coefficients, convergent validity, and ICC. The results mirrored findings from other samples, but all items exhibited misfit in a unidimensional model. 1. Four factors were identified through EFA and CFA. Yet, items 3 and 7 were deemed misfitting in Rasch PCM and RSM analysis. Consequently, these items were excluded from the original 20-item scale. The initial evaluation of person fits revealed that the lack of unidimensionality assumption led to the misfit. However, eliminating respondents with diverse patterns did not enhance the overall fit indices. To sum up, all aforementioned items displayed misfit in a unidimensional population. Additionally, unidimensionality assumes the scale measures a single underlying construct. Unidimensionality refers to the ability to measure a single concept, trait, or attribute. For instance, a personality scale, attitude scale, or any other scale that is unidimensional would consist of items that are solely related to the specific concept being measured.

Moreover, out of the 20 items, two had category fit statistics greater than 1.5, despite the fact that the average measures and step calibrations for all five response categories were consistently increasing. According to Linacre, achieving a minimum of 1.0 logits between step calibrations is crucial for a three-category scale to have the optimal number of response categories [14]. In addition, there were no significant differences between older men and women in the sum score of OFAKS and its four domains (*P* ≤ 0.05).

The results revealed that the satisfactory model for the scale has a 4-factor structure that accounts for 73.51% of the variance, based on exploratory and confirmatory factor analyses. Further, McDonald's omega and Cronbach's alpha were both higher than 0.7 for the 18-item scale and four factors. The study also indicated that the 18-item version of OFAKS has improved in convergent rationality and internal consistency with the new 4-factor structure. The number of response categories requires evaluating all versions, according to Rasch's analysis results, though the PCM and RSM designated that all the items contributed satisfactorily to their domain [21]. Based on the results of the likelihood ratio tests, there were no significant differences in model fit between MI models [22, 23]. This indicates that the OFAKS version was equally effective for both men and women. Additionally, as an official language of Iran, the Persian version of the OFAKS questionnaire was administered to the adult participants who shared a similar ethnicity. Based on the psychometric properties and IRT characteristics, it can be concluded that the 18-item version of OFAKS is superior, although further research is required.

## 5. Limitations and Suggestions

This study had some limitations, such as difficulty reaching inhabitants in certain areas of the south and southwest of Iran due to geographic dispersion. The survey was conducted by native questioners from the same areas. Bilingualism was also a concern, as the Qashqai Turks and Lors have their own languages. Therefore, questioners were selected who were familiar with the local language of the area. Additionally, since the data collected were from aged couples who were caring for their grandchildren, further investigation is required on single grandfathers or grandmothers.

Due to its cultural sensitivity, the 18-item OFAKS is recommended as a reliable and valid multidimensional instrument to be used among middle-aged grandparents in Iran and other societies with similar cultures in the MENA region. Future research should focus on examining the expressive aspects of various psychological constructs and develop culturally sensitive items to represent them. Since there is no criterion scale available as a golden standard for criterion-related validity, ROC analysis was not conducted in this study, and the cutoff point for the OFAKS could not be determined. It is also suggested that future validation studies evaluate the OFAKS's versions and response categories, particularly among never-married and widowed men and women who care for their siblings' children. In Iran, there is a need to take into account the gender and cultural aspects when considering the awareness of first aid among grandparents. Grandparents have a significant impact on the growth and emotional well-being of young children, as they introduce them to family history and provide support for their development. Studies indicate that grandmothers who actively care for their grandchildren experience better physical and mental health compared to those who do not. Consequently, the presence of grandchildren with grandparents is crucial, necessitating the availability of emergency supplies such as a first aid kit in case of unforeseen incidents.

## Figures and Tables

**Table 1:** Results of EFA (*n* = 285) and CFA (*n* = 200) performed on the OFAKS (18 items).

Items	Mean	SD	IQR	Factor loadings (95% CI)				*P* ^∗^	Uniqueness (Psi)
				Factor 1 (KNWL.)	Factor 2 (ATT.)	Factor 3 (BARR.)	Factor 4 (INT.)		
1	0.920	0.357	0.228	0.476				<0.001	0.76715
2	0.847	0.362	0.431	0.558				<0.001	0.64786
3	**0.207**	**0.406**	**0.165**	—				**0.651**	**0.13575**
4	0.633	0.484	0.234	0.351				<0.001	0.86242
5	0.607	0.490	0.240	0.301				0.027	0.88847
6	0.387	0.489	0.239	0.331				0.001	0.87572
7	**0.227**	**0.420**	**0.176**	—				**0.474**	**0.12581**
8	0.793	0.406	0.465	0.380				0.014	0.87512
9	0.727	0.933	0.871		0.360			0.015	0.91695
10	0.927	0.977	0.954		0.880			<0.001	0.56507
11	0.333	0.620	0.385		0.591			<0.001	0.63847
12	0.760	0.939	0.882		0.904			<0.001	0.45613
13	0.767	0.937	0.878		0.893			<0.001	0.46979
14	0.227	0.520	0.270			0.331		0.008	0.90304
15	0.200	0.518	0.268			0.683		<0.001	0.54104
16	0.153	0.488	0.238			0.874		<0.001	0.42395
17	0.201	0.543	0.295			0.505		<0.001	0.74215
18	0.280	0.603	0.364			0.356		0.025	0.90762
19	1.320	0.892	0.796				0.989	0.009	0.51501
20	1.400	0.695	0.483				0.749	0.003	0.50992
Factor determinacy index				1	0.924	0.912	0.914		
McDonald's omega				0.687	0.684	0.675	0.681		
Cronbach's *α*				0.694	0.688	0.669	0.687		

Parameter values (5,000 bootstrap 95% CI)									

Eigenvalue	74.11								
Variance explained (%)	73.51								
Keiser–Meier–Olkin (KMO) test (CI 95%)	0.846 (0.640–0.912)								
Bartlett's test of sphericity (BTS)	924.4 (Chi sqr/df = 1.294 m df = 210; *P*=0.001)								
Extraction method	Maximum likelihood (ML) with 11 varimax rotations								
Schwarz's Bayesian information criterion (BIC)	4358								
Akaike information criterion (AIC)	4178								
Conditional Akaike information criterion (CAIC)	4648								
Comparative fit index (CFI)	0.951								
Goodness of fit index (GFI)	0.958								
Tucker–Lewis index (TLI)	0.942								
Standardized root mean squared residual (SRMR)	0.0724 (0.0135–0.0804)								
Root mean square error (RMSEA)	0.0444 (0.0213–0.0625)								
Factor determinacy index (FDI)	0.937								
Cronbach's *α*	0.655								
McDonald's omega	0.645								
Δ McDonald's omega	0.694								

*Note*. Items 3 and 7 have been omitted to all versions according to the EFA, CFA, and IRT results. Significant loadings ≥0.3 are shown. F1, F2, F3, and F4 were named as KNWL = knowledge, ATT = attitude, BARR = barriers, INT = ntention. ^∗^Significant test (*Z*) in the CFA model <0.05. The item is not statistically significant, *P* < 0.05.

**Table 2:** IRT psychiatric analysis in the polytomous Rasch model for the OFAKS 18 items (RSM and PCM models).

Items	RSM measures^a^		Infit	Outfit	Delta-tau	Thurstone thresholds of the PCM		
	Measure	S.E.				1	2	3
1	1.5212	0.208	0.617	0.409	0.444	1.30	1.70	1.81
2	0.5451	0.115	0.286	0.338	0.455	1.30	1.70	1.81
3	**3.2117**	**0.184**	**1.809**	**1.940**	**0.985**	**2.02**	**2.70**	**2.81**
4	0.1048	0.121	0.540	0.619	0.474	1.30	1.70	1.81
5	0.0454	0.123	0.636	0.696	0.415	1.30	1.70	1.81
6	0.5206	0.142	0.604	0.663	0.408	1.30	1.70	1.81
7	**2.1138**	**0.177**	**0.814**	**0.882**	**0.927**	**2.07**	**2.70**	**2.81**
8	0.4393	0.116	0.423	0.494	0.467	1.30	1.70	1.81
9	0.3037	0.117	1.513	1.482	0.438	1.30	1.70	1.81
10	0.7010	0.114	1.066	1.008	0.429	1.30	1.70	1.81
11	0.6914	0.150	0.959	0.783	0.453	1.30	1.70	1.81
12	0.3721	0.116	1.054	0.963	0.439	1.30	1.70	1.81
13	0.3856	0.116	1.038	0.957	0.425	1.30	1.70	1.81
14	1.1138	0.177	1.208	1.095	0.478	1.30	1.70	1.81
15	1.2463	0.187	1.387	1.525	0.464	1.30	1.70	1.81
16	1.5226	0.212	1.501	1.512	0.468	1.30	1.70	1.81
17	1.2463	0.187	1.407	1.579	0.461	1.30	1.70	1.81
18	0.8855	0.162	1.492	1.532	0.471	1.30	1.70	1.81
19	1.4792	0.119	1.501	1.404	0.452	1.30	1.70	1.81
20	1.6546	0.123	0.847	0.834	0.444	1.30	1.70	1.81

*Note.* Infit = information-weighted mean square statistic; Outfit = outlier-sensitive means square statistic. ^a^Model Fit statistics for 18 items: person reliability = 0.676, MADaQ3 = 0.082, AIC = 4479, BIC = 4606, item separation index = 8.32 (suggested cutoff >2), person separation reliability = 0.84 (suggested cutoff >0.7), item separation reliability = 0.91 (suggested cutoff >0.7), person separation index = 2.32 (suggested cutoff >2), *P* < 0.001. The item is out of threshold (0.5–1.5) and misfitted.

**Table 3:** Results of measurement invariance for the OFAKS (18 items) between older women and men (*n* = 485).

Model	*χ* ^2^	*df*	*P*	CFI	GFI	SRMR	RMSEA	ΔCFI	ΔGFI	ΔSRMR	ΔRMSEA	∆*χ*^2^	Δdf	Δ*P*
Configural	221.23	482	<0.001	0.951	0.959	0.01	0.034	—	—	—	—	—	—	—
Thresholds	232.11	482	<0.001	0.957	0.961	0.01	0.034	0.000	0.0001	0.0001	−0.002	418.05	471	>0.99
Loadings	233.47	482	<0.001	0.957	0.960	0.01	0.034	0.000	0.0001	0.0001	−0.002	21.13	95	>0.99

*Note.* The configural model always serves as a reference. *CFA*, confirmatory factor analysis, *CFI*, comparative fit index, ΔCFI, difference in CFI; *df*, degrees of freedom; Δdf, difference in df; *p*, statistical significance of *χ*^2^; Δp, statistical significance of ∆*χ*^2^; *RMSEA*, root mean square of approximation; ΔRMSEA, difference in RMSEA; *SRMR*, standard root mean square residual; ΔSRMR, difference in SRMR; *GFI*, goodness of fit index; ΔGFI, difference in GFI; *χ*^2^, overall scaled chi-square statistic; ∆*χ*^2^, scaled chi-square difference statistic.

**Table 4:** The Pearson correlation coefficient between total score of OFAKS (18 items) and its domains (*n* = 485).

	Knowledge	Attitude	Barriers	Intention
Knowledge	—			
Attitude	0.442^∗∗^	—		
Etiology	0.838^∗^	0.865^∗^	—	
Intention	0.454^∗^	0.413^∗^	0.509^∗^	—
OFAKS	0.618^∗∗^	0.841^∗∗^	0.649^∗∗^	0.681^∗∗^

^∗^Correlation is significant at the 0.05 level (2-tailed). ^∗∗^Correlation is significant at the 0.01 level (2-tailed). *Note.* OFAKS = total score of scale.

## Data Availability

The data that support the findings of this study are available from the corresponding author upon reasonable request.

## Additional Points

*Human & Animal Rights*. No animals were used for studies that are the basis of this research. This research was conducted on humans are in accordance with the Helsinki Declaration of 1975, as revised in 2013 (https://ethics.iit.edu/ecodes/node/3931). *The Standard for Reporting.* The STROBE guidelines and methodology and COSMIN checklist were followed during this study. *What This Paper Adds.* (i) Classic factor analysis (EFA and CFA) approved OFAKS. (ii) The OFAKS is more reliable among Iranian samples. (iii) The IRT analysis models (PCM & RSM) approved OFAKS too. *Applications of Study Findings*. (i) A practical tool for measuring first aid knowledge of older adults. (ii) Evaluating attitudes and barriers of participating in the first aid education. (iii) It uses in community-based settings as health policy making.

## Supplementary Materials

Supplementary Materials The supplementary file contains the completed and developed version of the OFAKS, which consists of 18 items following the application of psychometric analyses based on the classical model and the IRT method. The findings indicate that items 3, 4, and 7 are recommended for removal from the questionnaire. Additionally, a Persian version of this instrument is accessible for the Persian-speaking populations in Iran, Afghanistan, Tajikistan, and the Central Asian countries.
