# Supplementary material for: Psychometric Properties under EFA, CFA, Measurement Invariance, and IRT Models for Older Adults' First Aids Knowledge Scale among Iranian Grandparents: The Modified Scale
Source: ScientificWorldJournal. 2024 Aug 26;2024:6208571. doi: 10.1155/2024/6208571 (PMC11368547; doi:10.1155/2024/6208571)
Supplement: Supplementary Materials — The supplementary file contains the completed and developed version of the OFAKS, which consists of 18 items following the application of psychometric analyses based on the classical model and the IRT method. The findings indicate that items 3, 4, and 7 are recommended for removal from the questionnaire. Additionally, a Persian version of this instrument is accessible for the Persian-speaking populations in Iran, Afghanistan, Tajikistan, and the Central Asian countries. [file 6208571.f1.doc]

**Older Adults' First Aid Knowledge Scale (OFAKS, 18-item)**

**Dear Participant;**

Gender: Male 🞏 Female 🞏 / Years of Age: .................

Greetings, considering the importance of first aid knowledge, the following questionnaire has been prepared. While thanking you for your cooperation, please read the questions carefully and mark your answer with⌧. Your answers will be completely confidential and participation is voluntary, also, anonymity is guaranteed.

**Section Knowledge**

**1- Please assess your knowledge of first aid (both theoretical and practical) on the following scale from 0 to 5 with.** (Zero means that you have no knowledge of first aid and 5 means that you know both theory and practice of first aid very well.)

| I don't know at all. [0] | I have little information. [1] | I know. [2] | | | |
| --- | --- | --- | --- | --- | --- |
| 0 | 1 | 2 | 3 | 4 | 5 |

**2- Which phone number do you call if you need urgent medical help?**

🞏 115 [1] 🞏 110 [0] 🞏 I don't know. [0]

**~~3- In cardiopulmonary resuscitation; what is the correct ratio of chest compressions and artificial respiration in an adult?~~ ^~~a~~^**

~~🞏 15 chest compressions and 2 artificial breaths [0]~~

~~🞏 30 chest compressions and 2 artificial breaths [1]~~

~~🞏 I don't know. [0]~~

**4- Please answer the following statement with "yes" for the correct sentence and "no" for the wrong sentence.**

If someone starts coughing while eating (due to airway obstruction), we should encourage them to keep coughing.

🞏 Yes [1] 🞏 No [0] 🞏 I don't know. [0]

- Please mark the true or false statement in the following sentences with.

| **Items** | **True** | **False** | **I don't know** |
| --- | --- | --- | --- |
| **5- We stop severe bleeding by the direct pressure on the bleeding site.** | **[1]** | **[0]** | **[0]** |
| **6- At the time of amputation, the amputated limb is NOT placed directly on the ice.** | **[1]** | **[0]** | **[0]** |
| **~~7- If bones and joints are damaged, a person should not eat or drink.~~ ^~~a~~^** | **[1]** | **[0]** | **[0]** |
| **8- After a hip fracture, the injured person should be taken to a medical center.** | **[1]** | **[0]** | **[0]** |

**Section Attitude**

**9- When someone needs first aid on the street. Are you willing to help him or ask others for help?**

🞏 I would give first aid myself. [2] 🞏 I would ask others for help. [0] 🞏 I don't know. [1]

**10- Have you ever completed first aid training or acquired first aid knowledge in another way?**

🞏 Yes [2] 🞏 No [0] 🞏 I don't know. [1]

- Please answer to items 11 and 12, if item 10 is YES.

**11- How many years have passed since your last first aid training?**

🞏 1 year or less [2] 🞏 Between 2 and 4 years [1] 🞏 More than 5 years [0]

**12- Do you need to repeat your first aid course?**

🞏 Yes [2] 🞏 No [0] 🞏 I don't know. [1]

- Please answer to items 13, if item 10 is NO.

**13- Do you need to learn first aid?**

🞏 Yes [2] 🞏 No [0] 🞏 I don't know. [1]

**Section Barriers**

- Why haven't you participated in the first aid course yet or haven't repeated it?

| **Barriers** | **Agree** | **Disagree** | **I do not know.** |
| --- | --- | --- | --- |
| **14- Cost: The first aid course is not cheap.** | **[0]** | **[2]** | **[1]** |
| **15- Ability: I cannot participate in these courses** | **[0]** | **[2]** | **[1]** |
| **16- Time: I don't have time to participate in these courses.** | **[0]** | **[2]** | **[1]** |
| **17- Holding: because there are no such courses for the elderly.** | **[0]** | **[2]** | **[1]** |
| **18- Interest: because I have no interest at the moment.** | **[0]** | **[2]** | **[1]** |

**Section Intention**

**19- I participate in the first aid course this year.**

🞏 Yes [2] 🞏 No [0] 🞏 I don't know. [1]

**20- I participate in the first aid course with my spouse/children/family/ relatives this year.**

🞏 Yes [2] 🞏 No [0] 🞏 I don't know. [1]

Thanks for your time and answers.

***Note***: The numbers inside [ ] are the score coefficient of that item.

a. The strikethrough sentences are the items omitted in the final psychometric results.

***Ethical Approval Code:*** *IR.SUMS.SCHEANUT.REC.1402.009 on March 5, 2023.*
